# Electrochemical Characterization and Detection of Lead in Water Using SPCE Modified with BiONPs/PANI

**DOI:** 10.3390/nano11051294

**Published:** 2021-05-14

**Authors:** Enyioma C. Okpara, Samuel Che Nde, Omolola E. Fayemi, Eno E. Ebenso

**Affiliations:** 1 Department of Chemistry, School of Physical and Chemical Sciences, Faculty of Natural and Agricultural Sciences, North-West University (Mafikeng Campus), Private Bag X2046, Mmabatho 2735, South Africa; ebrochima@gmail.com; 2 Material Science Innovation and Modelling (MaSIM) Research Focus Area, Faculty of Natural and Agricultural Sciences, North-West University (Mafikeng Campus), Private Bag X2046, Mmabatho 2735, South Africa; 3 Department of Geography and Environmental Sciences, North-West University, Mafikeng Campus, Mmabatho 2735, South Africa; Samuel.Nde@nwu.ac.za; 4 Institute of Nanotechnology and Water Sustainability, College of Science, Engineering and Technology, University of South Africa, Johannesburg 1710, South Africa; eno.ebenso@gmail.com

**Keywords:** green waste management, citrus peels extracts, screen plate electrodes, heavy metals, bi-metallic oxide nanoparticles, environmental monitoring, electrochemical detection

## Abstract

The need for constant assessment of river water qualities for both aquatic and other biological survival has emerged a top priority, due to increasing exposure to industrial pollutants. A disposable screen print carbon electrode was modified with a conductive polymer (PANI) and Zn and/or Cu oxides NPs, obtained through bioreduction in citrus peel extracts (lemon and orange), for ultra-sensitive detection of PB^2+^, in the Crocodile River water sample. The synthesized materials were characterized with Fourier-transform infra-red spectroscopy (FTIR), ultra-violet visible spectroscopy (UV-Vis), and scanning electron microscopy (SEM). The SPC-modified electrodes designated as SPCE/LPE/BiONPs/PANI and SPCE/OPE/BiONPs/PANI were characterized using cyclic voltammetry (CV) and electrochemical impedance spectroscopy (EIS) and eventually deployed in the electrochemical detection of PB^2+^ in water using square wave voltammetry (SWV) technique. The electrochemical responses of the modified electrodes for both CV and EIS in 0.1 M HCl demonstrated enhanced performance relative to the bare SPCE. A detection and quantification limit of 0.494 ppb and 1.647 were obtained at SPCE/LPE/BiONPs/PANI, respectively, while a detection and quantification limit of 2.79 ppb and 8.91 ppb, respectively, were derived from SPCE/OPE/BiONPs/PANI. The relative standard deviations (RSD) for SPC electrode at a 6.04 µM PB^2+^ analyte concentration was 4.76% and 0.98% at SPCE/LPE/BiONPs/PANI and SPCE/LPE/BiONPs/PANI, respectively. The effect of copper, zinc, iron, cobalt, nickel, and magnesium on the stripping peaks of PB^2+^ at SPCE/OPE/BiONPs/PANI, showed no significant change except for cobalt, with about 17.67% peak current drop. The sensors were assessed for possible determination of PB^2+^ in spiked river water samples. The average percentage recovery and RSD calculated were 94.25% and 3.74% (*n* = 3) at SPCE/LPE/BiONPs/PANI and, 96.70% and 3.71% (*n* = 3) at SPCE/OPE/BiONPs/PANI, respectively. Therefore, the fabricated sensor material could be used for environmental assessment of this highly toxic heavy metal in the aquatic system

## 1. Introduction

Water is essential to living and plays vital roles in countless human activities. Globally, there has been an increasing demand for potable water both for industrial and commercial purpose. However, rapid growth in population and attendant industrial growth in developing economies have emerged as key drivers into a global water scarcity. Based on the existing population growth rate, by the year 2050, the population of the world is projected to get close to 9 billion and the magnitude of the water crisis will keep rising [1].

Naturally sourced water is, as a rule, largely unsuitable to be used without any form of treatment, particularly for consumption, a sequel to the exposure to a range of contaminants, which could be organic, inorganic, pathogenic, etc. [2,3,4,5]. It is projected that 3.2% of global deaths could be attributed to unsafe water resulting from poor hygiene and sanitation [6] and four-fifths of the diseases in humans globally are as a result of consumption of contaminated water, acute hygiene deficiency and inadequate sanitary knowledge [7].

Currently, of the water contaminants, heavy metal pollution is fast becoming a serious environmental concern due to their increasing outlets, namely electroplating, mining, chemical plants, metallurgy, household, and agriculture wastewater in the modern society [8,9]. Additionally, heavy metals exposure, even in trace amounts, could pose great risk to humans, exerting harmful effects on the environment and other ecological receptors [10,11,12,13,14]. The environmental monitoring and control of exposure is consequently of great importance and has drawn much attention in many research frontiers. However, the detection of heavy metals is a complicated procedure, often involving widespread processes of preparation of sample leading to the measurement, when analytical techniques are employed [15]. Such standard techniques for the detection of heavy metal ions such as inductively coupled plasma optical emission spectrometry (ICP-OES) [16,17,18], UV–Vis spectrometry, [19,20,21] atomic absorption/emission spectroscopy [22,23,24] and laser-induced breakdown spectroscopy (LIBS) [25,26], would not be suitable for in situ, fast, easy and low-cost operation.

The growing and mandatory need for real-time on-site tracking of quality of water and the environment necessitates an alternative reliable and sensitive technique which is affordable and exerts less pressure on the environment [27]. The electrochemical monitoring technique has emerged that promising platform for handy, affordable substitute with high selectivity and a low limit of detection [28,29]. However, the choice of applicable electrochemical sensors in environmental assessment of heavy metals, using various electrochemical techniques could undermine their applicability [30]. The most effective and reliable electrode materials are reported hybrid materials, incorporating various precursors with diverse complementary and supplementary properties [31,32,33]. Following the development of screen printing technology, mass production of screen-printed electrodes (SPEs) has enjoyed extensive achievement [34]. SPEs share similar electrochemical properties with traditional bulk electrodes and possess the superiority of on-site sensing ability, real-time quantification, and ease of operation over the bulk electrodes.

Generally, electrodes modified with nanoparticles (NPs) have attracted huge interest owing to their exceptional chemical, electronic, and physical properties, which no other materials for sensing can attain or the equivalent counterpart of the bulk materials. Using DPASV, Honeychurch and co-workers employed unmodified SPCEs in detection of lead [35,36] in pond waters and copper [36] in waters and serum. The LOD calculated were 2.5 ppb and 8.2 ppb, respectively. Using the SWASV, Guell et al. [37] detected simultaneously cadmium and lead in seawater with the SPCEs. They realized an LOD of 2.9 ppb and 1.8 ppb for deposition time of 120 s. Aragay et al. [38] examined the temperature dependence of electrochemical stripping activity of a HM sensor using nanoparticles of carbon. They realized a multiple detection of copper, lead, mercury, and cadmium ions with higher efficiency, as a result of the availability of larger surface area for the metal deposition and electrons transfer enhanced as a sequel to the increase in edge-like planes. Sommers et al., previously employed polyaniline (PANI), polyanilinepoly (2,2′–dithiodianiline) (PANI-PDTDA) or PANI-methylene blue [39,40,41] in detecting metals (mercury, nickel, lead, and cadmium), realizing a LOD of for 56 ppb for mercury after 120 s deposition time using DPASV as EC techniques.

In this work, the unique properties of environmentally friendly and low-cost bicomponent mixture of CuO and ZnO, named hereinafter bioxide NPs (BIONPs) and PANI blend, were employed in the modification of SPCE for quantification of lead ion in Crocodile River, South Africa, close to a point surrounded by agricultural activities, using SWV technique.

## 2. Materials and Methods

### 2.1. Materials

*Citrus limon* (Lemon) and *Citrus sinensis* (sweet orange) peels were precursor plant part used. Potassium hexacyanoferrate (III), (K_3_[Fe (CN)_6_]; 99% purity), zinc nitrate (Zn (NO_3_)_2_, and zinc acetate [(CH_3_.COO)_2_ Zn.2H_2_O all of which have analytical purity, being acquired from Sigma-Aldrich, (Chemie GmbH, Steinheim, Germany). Dimethyl sulfoxide (DMSO, (CH_3_)_2_SO; 99%), N, N-Dimethylformamide (DMF, HCON(CH_3_)_2_, Aniline (C_6_H_7_N; ≥99% purity), and ortho-phosphoric acid (85%), and sodium hydroxide (NaOH; 99% purity), were bought from Emsure Iso, Merck KGaA, (Darmstadt, Germany). HCl (30%) of analytical purity was gotten from Promark Chemicals (Johannesburg, South Africa). Cupric nitrate tri-hydrate (Cu (N0_3_)_2_.3H_2_O and Zinc nitrate (Zn (NO_3_)_2_ of analytical purity was purchased from SAARCHEM pty ltd (Gauteng, South Africa) and, Sigma-Aldrich (St. Louis, MO, USA), respectively. Potassium chloride (KCl; 3 mol/L) electrolyte was acquired from Metrohm Ltd., (cH-9100 Herisau, Ionenstrasse, Switzerland) and used in the electrochemical studies. Distilled and deionized water (Table 1) were obtained from the laboratory water from purite and vent filter MPKO1, Millipore S.A.S 67,120 (Molsheim, France), respectively. Nitric acid, (HNO_3_, 55%) was purchased from SAAR, Friestand Drive, Modderfontein, Gauteng, 1645 South Africa. Cadmium (II) chloride (CdCl_2_; ≥99%), mercury (II) chloride (HgCl_2;_), and lead nitrate, (Pb(NO_3_)_2_; 99.5%), were gotten from Fluker, Sigma-Aldrich, 3050Spruce Street, St Louis, MO, 63103, USA, edutrade, (Gaunteng, South Africa), and, Glassworld, Robertville, Johannesburg, South Africa, respectively.. Ammonium persulphate (APS; (NH_4_)_2_S_2_O_8_) was acquired from Sigma-Aldrich (St. Louis, MO, USA). In total, 0.1 M buffer solution of the probe were prepared using K_3_[Fe(CN)_6_] and KCl. Then, 0.1 M solution each of HCl and NaOH were employed to adjust the value of the pH of the buffer solution. However, 1 M NaOH was used in the optimization of the pH for the electrochemical detection of the analytes to reduce dilution of the solution.

### 2.2. Material Characterization

The materials were characterized using, Carry 300, UV-Vis Spectrophotometer, Agilent Technologies, Germany, spectroquant Prove300, Merck KGaA, (Darmstadt, Germany), and UV–Vis Uviline 9400 (Sl Analytics, Hattenbergstr.10, D-55122 Mainz, Germany) in the investigation of the optical properties of the nanomaterial fabricated. FTIR (Opus Alpha-P, Brucker Corporation, Billerica, MA, USA) being employed in the evaluation of the functional groups that intermingled with the metal salts and hence performed the role of a reducing or capping agent or both. Quanta FEG 250 ESEM, (ThermoFisher Scientific, Hillsboro, OR, USA) operating on an acceleration voltage of 15.0 kV was employed to describe the surface structure of the nanomaterials prepared. DropSense (analysed with Metrohm Dropview 200 and 8400 series), incorporated with screen plate electrode (SPE) having a 4 mm carbon working electrode (WE) diameter at its centre, carbon counter electrode (CE) and an Ag pseudo-reference (Ag/AgCl) electrode (RE) was used in electrochemical studies. A potential window of −1000 mV to 1200 mV, was employed in the cyclic voltammetry study, using a common scan step of 10 mV/s. The same range of windows was also used in the electrochemical detection of the target Pb^2+^ metal.

### 2.3. Synthesis of Bioxide NPs

The bioxide NPs were prepared by simple bioreduction method using citrus peels extracts in an alkaline medium. The preparation of the orange and lemon peels extract used (OPE and LPE, respectively), have been detailed in our previous works [30,42]. In total, 0.1 M (100 mL) each of the precursors salts, Cu(NO_3_)_2_ and Zn(NO_3_)_2_ were added at the same time into two different conical flask of appropriate volume and heated above 90 °C under a magnetic stirring. Then, 20 mL peel extracts from lemon and orange were added each into the different conical flasks containing the equal ratio of the precursors. Colour changes were noted for any possible bireduction within the first 10 min and followed up with UV-Vis run. After 10 min, about 5–10 mL 1 M solution of NaOH was introduced into each the mixture under vigorous stirring. Immediately, the colour of the mixtures changed from bright light blue through series of colour changes to whitish yellow, indicating the bioreduction of both Zn and Cu. Another sample was collected for UV-Vis confirmation and subsequently the routine was repeated at hourly interval, while trying to keep the reactions conditions constant. After 3 h, the mixtures were cooled down, with thick pale whitish black precipitate settling immediately at the base of the conical flask. The separate mixtures were later centrifuged each for 15 min at 6000× *g* rpm, and washed thoroughly at least twice with distilled water for another 5 min at the same speed, leaving the precipitate settling at the base. The samples were collected, and oven dried above 200 °C for more than 5 h in a thoroughly washed crucible. The dry samples were collected and kept in airtight vessels for further use.

### 2.4. Synthesis of PANI

A total of 0.5 g aniline was added to 15 mL of 1 M HCl solution at room temperature and stirred over a magnetic stirrer, and the mixture was cooled down to <5 °C in an ice bath. Then, 1.28 g of APS in another 15 mL of 1 M HCl was cooled to <5 °C was added stepwise to the aniline solution, then stirred at such temperature range, in an ice bath for 4 h, resulting in the formation of green precipitate. The mixture was allowed to settle overnight and thoroughly washed with deionized water. The precipitate was dried in the oven at 50 °C for 24 h. To get de-doped polymer, the dried polymer powder was introduced into 50 mL of IM aqueous ammonium solution and mixed over a stirrer for 24 h at room temperature. The resultant wet precipitate was filtered, thoroughly washed using distilled water, and dried with the oven at 50 °C for about 48 h to get obtain emeraldine base, denoted as de-doped PANI hereafter.

### 2.5. Modification of the SPCE

Drop dry approach was used to treat each of the SPCE (disposable) modified. About 2 mg of the as prepared metal oxide NPs (MO NPs), and metal oxide NPs and PANI blend (MO NPs/PANI), were each dissolved in about 3–6 drops of DMSO and sonicated at room temperature. Typically, 20–30 µL of the resultant MO NPs, and MO NPs/PANI mixtures were each cast separately on the carbon working electrode and air-dried overnight to obtain SPCE modified with MO NPs, named hereinafter SPCE/CPE/MO NPs and a second SPCE modified with MO NPs/PANI, named hereinafter SPCE/CPE/MO NPs/PANI. Working electrodes hence characterized were, (i) unmodified SPCE, otherwise called bare SPCE, (ii) LPE/ZnONPs modified SPCE, hence termed SPCE/LPE/ZnONPs, (iii) OPE/ZnO NPs modified SPCE, designated hence as SPCE/OPE/ZnONPs, (iv) LPE/CuO NPs modified SPCE named hereinafter SPCE/LPE/CuONPs, (iv) OPE/CuONPs modified SPCE named hereinafter SPCE/OPE/CuONPs, (v) LPE/BIONPs modified SPCE named hereinafter SPCE/LPE/BIONPs, (vi) OPE/BIONPs modified SPCE named hereinafter SPCE/OPE/BIONPs, (vii) PANI modified SPCE named hereinafter SPCE/PANI, (viii) LPE/BIO NPs/PANI modified SPCE named hereinafter SPCE/LPE/BIONPs/PANI and (ix) OPE/BIONPs/PANI modified SPCE termed henceforth SPCE/OPE/BIONPs-PANI.

### 2.6. Cyclic Voltammetric and Impedance Measurements (EIS)

The electrochemical responses of the engineered nanomaterials modified SPCE were subsequently studied using CV in 0.01 M K_e_Fe(CN)_6_ probe in 0.1 M KCl at a pH value adjusted to approximately 7.0. Further, CV and EIS behaviour of fabricated electrodes were investigated in the electrolytes (pH ≈ 1.0), prepared using distilled water. During each study, the solution of either the probe or the electrolyte (40–50 µL) was placed on the surface of the electrodes to cover just the CE, RE and WE and scanned using the Dropview 200 and 8400 model software. All experimentations were done at atmospheric room conditions. The methodical procedures were easy and straightforward.

### 2.7. Electrochemical Tracing of Heavy Metal Ions

Pre-concentration of the electrode was performed on the SPC electrodes in the electrolyte (0.1 M HCl) containing the target Pb^2+^ analyte at a defined concentration, optimally selected deposition potential, deposition time, frequency, amplitude and incremental step of 4 mV. In practical terms, the modified electrodes were cycled using CV for about 20 times and conditioned for not less than 600 s in the SWV mode with at least 10 scans, to reduce the background current. The analysis of Pb^2+^ was performed with square wave voltammetry (SWV), which was dependent on the signal stability. All experimental procedures and measurements were performed in most cases in triplets at room temperature, and no oxygen removal was involved. Each measurement comprised of three distinct steps: electrode conditioning, analytical and background scan. For each addition of the analyte in the electrolyte, the solution was bubbled over time to ensure proper mixing before each measurement. The different steps were automated using the Dropview software. Both peak heights and peak derivatives were used for quantification. The real sample analysis was done by spiking the river water in 0.1 M HCl with the measured solution of the analyte.

## 3. Results and Discussion

### 3.1. UV-Vis (Ultraviolent Visible Spectroscopy)

The appearance of two distinguishable peaks at around 360 nm and 470 nm for the colloidal ZnO NPs and CuO NPs for both CPEs, respectively, suggests the formation of a core-shell NPs [43,44,45]. This result agrees with the finding reported by Bayahia [46] and Bae [47]. The computed band gaps using Tauc plots for ZnO were 3.34 eV, and 3.30 eV for the OPE, and LPE BiONPs, respectively. These values are comparable with that of their monoxide counterparts [30,42]. Using the same Tauc plot, the computed band gap for CuO were 2.44 eV, and 2.45 eV and are consistent the band gap values of Cu_2_O reported in another work [48]. However, while the absorption peak (λ_max_) remained largely the same for both the monoxides NPs (MoONPs) and the BIONPs, the value of Eg was higher for the BiONPs than that of the MoONPs in each of their equivalent oxides. This suggests that the BIONPs will have reduced particle size in relation to their counterpart MoONPs as confirmed in the XRD analysis the subsequent sessions. This improvement in particle morphology is expected to bring some interesting contribution in the size dependent electrochemical behaviours of the engineered NPs.

**Figure 1:**
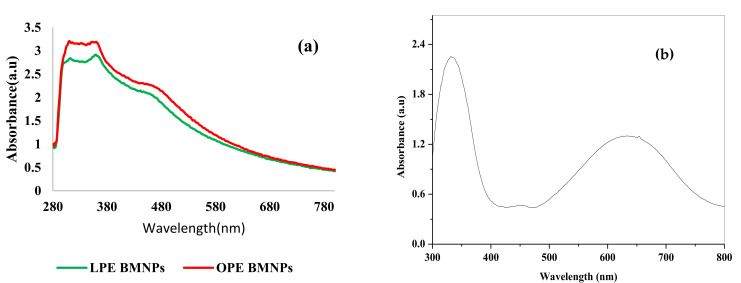
The UV-Vis of (**a**) Comparison of the synthesis for the two CPE/NPs (**b**) PANI.

The as-prepared PANI exhibited two distinct absorption bands at around 332 nm and 633 nm, corresponding to that of pure PANI (Figure 1). The λmax between 330 nm–360 nm, and 600–650 nm are typical of two chromophores, denoting oxidation states of emeraldine with y = 1, y = 0.5 [49]. The peaks of absorption, near 330 nm–360 nm is ascribed to the p–p* transitions in the aromatic rings. The absorption bands near 600–650 nm resemble that of the intramolecular electronic transitions between quinoid and benzenoid units. When PANI is doped with acids, the quinoid bands demonstrate hypochromism as presented in Figure 1, is suggestive of the existence of configuration of random coil typical of PANI salts. The representative low wavelength polaron bands near 400–440 nm as a result of the conductive nature of PANIs are typical of PANI salts only [50].

### 3.2. FTIR

**Figure 2:**
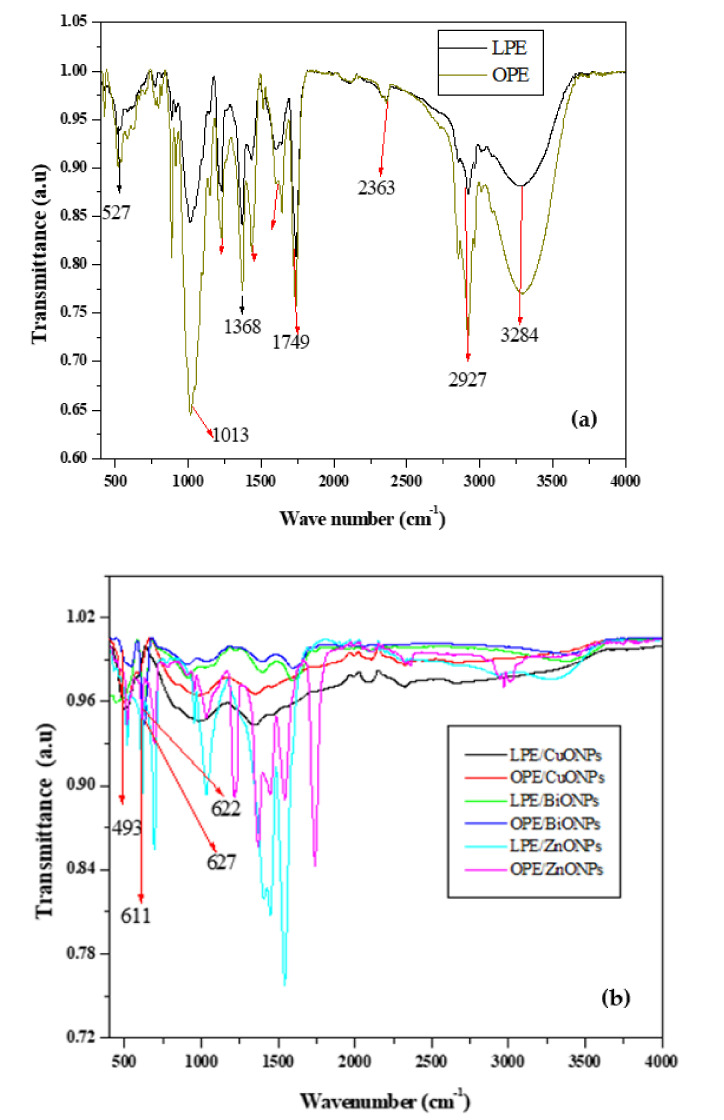
FTIR spectral of (**a**) citrus peels extract (CPE) and (**b**) CPE/NPs.

The FTIR of the citrus peels mediated nanoparticles (CPE/NPs) was carried out and presented (Figure 2a,b) to explore the phytochemicals responsible for the bioreduction, capping and effective stabilization of the composites of ZnO/CuO NPs. The results of FTIR indicate that the citrus peels contain bioactive compounds with functional groups corresponding to that of flavonoids, protein, phenols, dietary fibre and non-phenolic carotenoïds, monoterpenes, alcohols, esters and carbonyl compounds, limonene. [51]. The FTIR of the NPs reported, presents peaks corresponding to that of CuO and ZnO and the potential phytochemicals that could have been responsible for the effective reduction, capping and stabilization of the prepared CPE/NPs. The IR peaks in the region of 920 cm^−1^, 1013 cm^−1^, 1389 cm^−1^, 1600 cm^−1^ and 3284 cm^−1^, correspond to –CH out of plane bending vibration of trans or E-alkene, vibrations of carboxylic acids and bending frequencies of C–O, C–OH bending (in-plane), carbonyl stretches, and –OH stretching vibration on the surface of the bioxide NPs, respectively [52,53,54]. From the IR compared to that of the citrus peel, it is obvious that the functional group responsible for the three desired roles of bioreduction, capping and stabilization by electron donation, corresponds to that of flavonoids, proteins, esters, soluble sugars, carboxylic acid from phenolic acids, alcohol and carbonyl groups. A typical IR absorption bands for the OPE and LPE mediated NPs showed common strong peaks at 493 cm^−1^ for all CuONPs/CPE, typical of Cu-O stretching vibration [55,56] and diversified band of, 620 cm^−1^ for ZnO NPs of both BiONPs, 622 and 627 cm^−1^ for the, ZnO/LPE and ZnO/OPE, respectively, which is typical to that of ZnO band [57,58,59].

**Figure 3:**
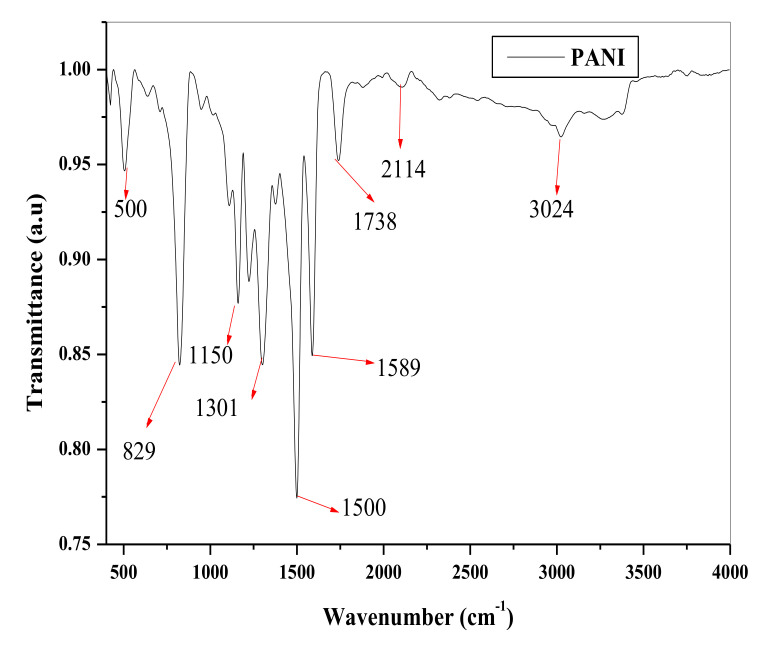
FTIR spectral of PANI.

The spectral evaluation of the FT-IR for the prepared PANI as seen in Figure 3 has shown that the vibrational peaks in the region between 3400–3200 cm^−1^ is the stretching vibration of N-H band, 3100–2800 cm^−1^ region corresponds to the C-H band, and broad band around 2800–2300 cm^−1^ specifies the existence of iminium planes with dopant ions on PANI matrix [60,61]. The typical vibrations in the regions between 1590–1560 cm^−1^ (1589 cm^−1^) and 1500–1490 cm^−1^ designated the presence of the of backbone of the PANI, springing from the stretching manners of the quinoid and the benzenoid rings [62,63]. The peaks close to 1300 cm^−1^ are representative of the stretching vibrations of strong C-N in PANI, while the peaks close to 1100–1160 cm^−1^ correspond to aromatic C-H in-plane deformation and prominent band close to 790–830 cm^−1^ is commonly attributed to the out-of-plane bending pattern of aromatic CAH groups in 1, 4-disubstituted aniline units [64,65,66,67]. This is indicative of the head-to-tail juxtaposition common with PANI. The peak around 500 cm^−1^ typifies another bending vibration of the backbone of PANI.

### 3.3. SEM

**Figure 4:**
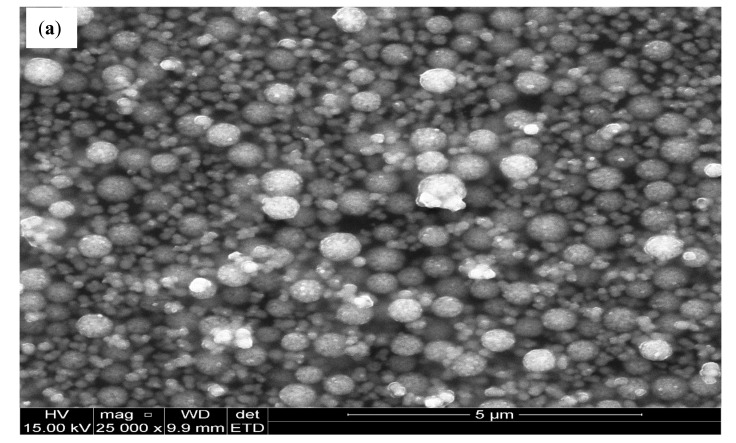

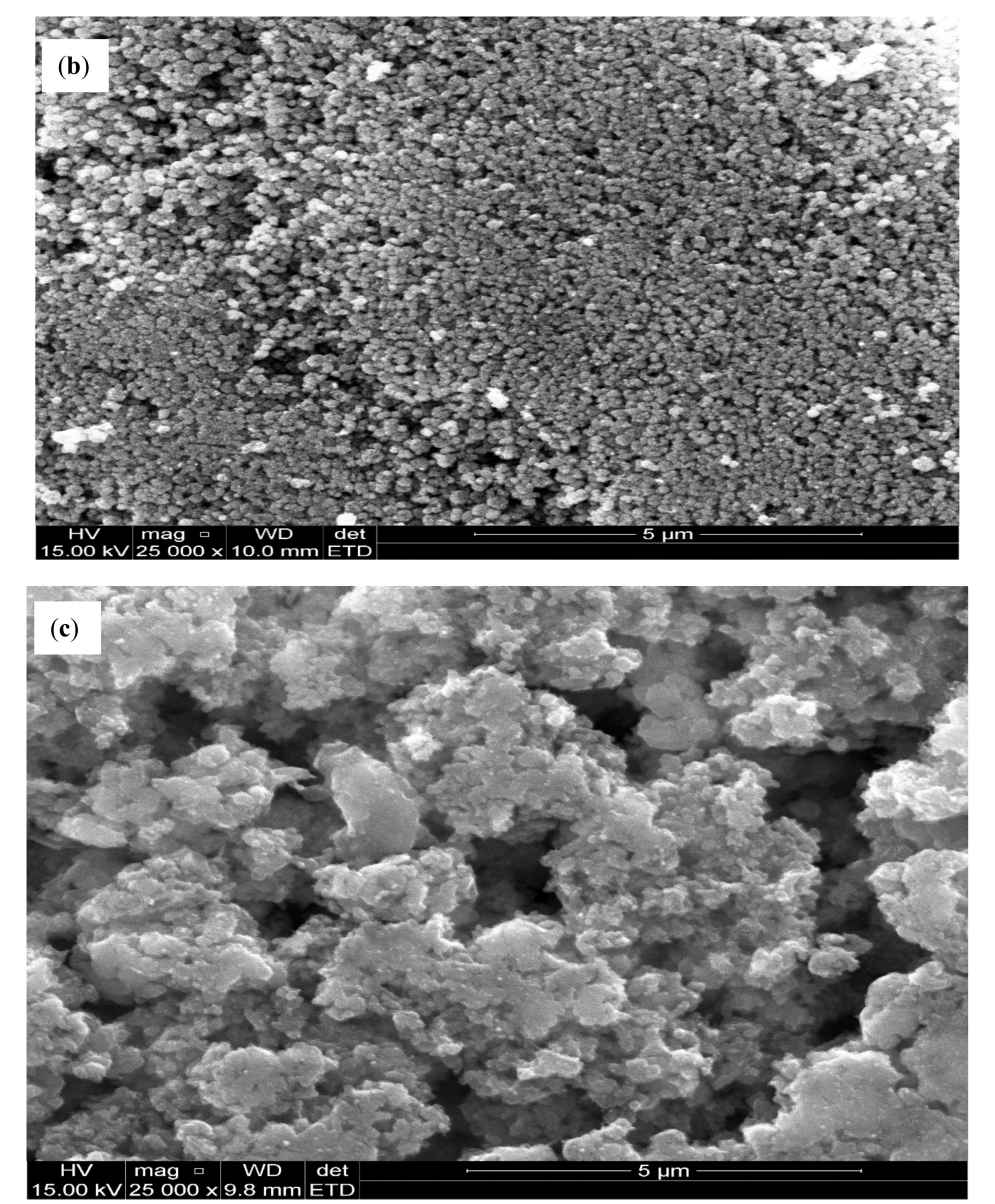
The SEM images of (**a**) LPE/BiONPs (**b**) OPE/BiONPs, (**c**), PANI.

The morphology of the synthesized NPs is shown in Figure 4. The LPE/BiONPs showed rough surfaced agglomerated balls of clusters that could result in smaller colloidal dimensioned NPs in appropriate solvents, while that of the OPE/BiONPs showed agglomerated spherical clusters having even surfaces, evidently devoid of cracks.

Figure 4 represents the surface morphology of the chemically prepared PANI, which indicated that the PANI has a coarse surface, which is similar to earlier literature reports [68,69]. The microstructure morphology of the PANI is considered as being of a positive effect on its particle specific surface area and the counter anions diffusion [68]; hence, it is profitable for the amplification of specific capacitance of PANI electrode. On the other hand, the rough surface may influence negatively the PANI’s conductivity [69].

### 3.4. Cyclic Voltammetry of the NPs Modified SPCE in 10 mM [Fe (CN)_6_]^4−^ Probe

**Figure 5:**
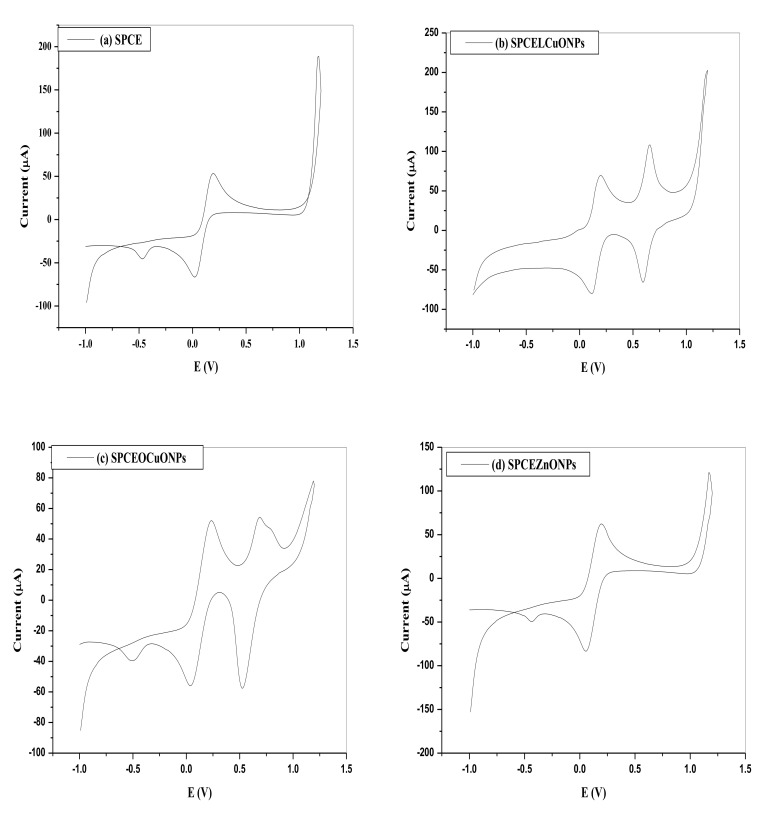

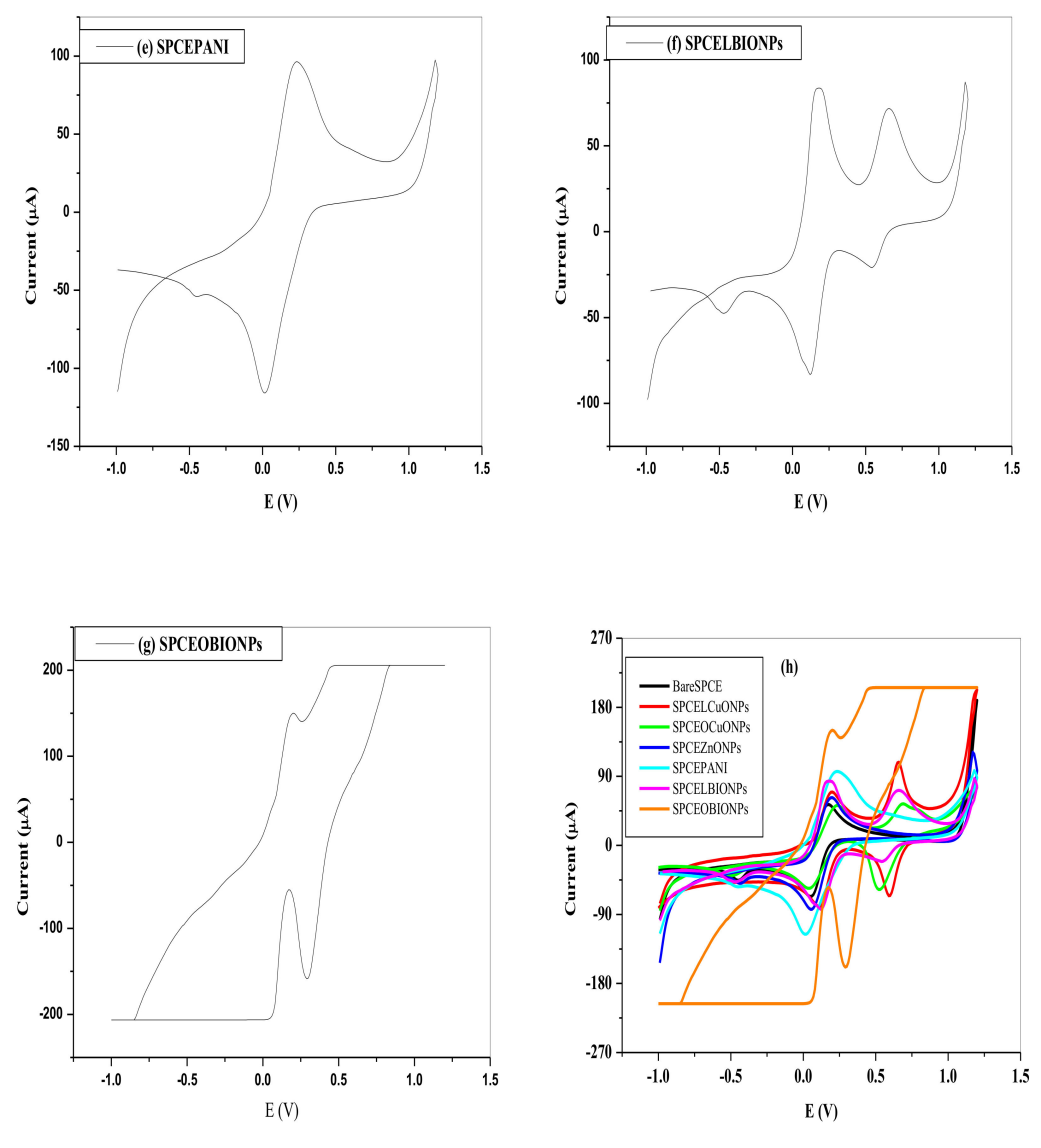
Individual and CV comparison of 0.01 M [Fe(CN)_6_]^4−^ in 0.1 M KCl at pH ≈ 7 on the (**a**) bare SPE, modified (**b**) SPCE/LPE/CuONPs (**c**) SPCE/OPE/CuONPs (**d**) SPCE/CPE/ZnONPs, (**e**) SPCE/PANI, (**f**) SPCE/LPE/BiONPs, (**g**) SPCE/OPE/BiONPs, and (**h**) modified SPCE (scan rate = 0.01 V/s, Potentials vs. Ag/AgCl).

The electrochemical responses of the SPCE and the nanoparticles modified SPCE, were explored. The individual and comparative voltammograms presented in Figure 5 reveals redox peaks at the bare SPE and all the modified electrodes that are well-defined. The additional redox peaks at 0.66 V, −0.59 V; 0.69 V, −0.53 V; and 0.66 V, −0.54 V for SPCE/LPE/CuO NPs, SPCE/OPE/CuO NPs and SPCE/OPE/BIO NPs could be attributed to the reduction of Cu^2+^ to Cu^0^ and oxidation of Cu^0^ to Cu^2+^, which underscores the effective amplification of the performance of the unmodified electrodes.

The peak anodic currents for the SPCE/LPE/CuONPs, SPCE/OPE/CuONPs, SPCE/CPE/ZnO NPs, SPCE/PANI, SPCE/LPE/BIONPs and SPCE/OPE/BIONPs are approximately 1.31, 1.0, and 1.16, 1.79, 1.56 and 2.78 times greater than the bare SPCE, respectively.

**Figure 6:**
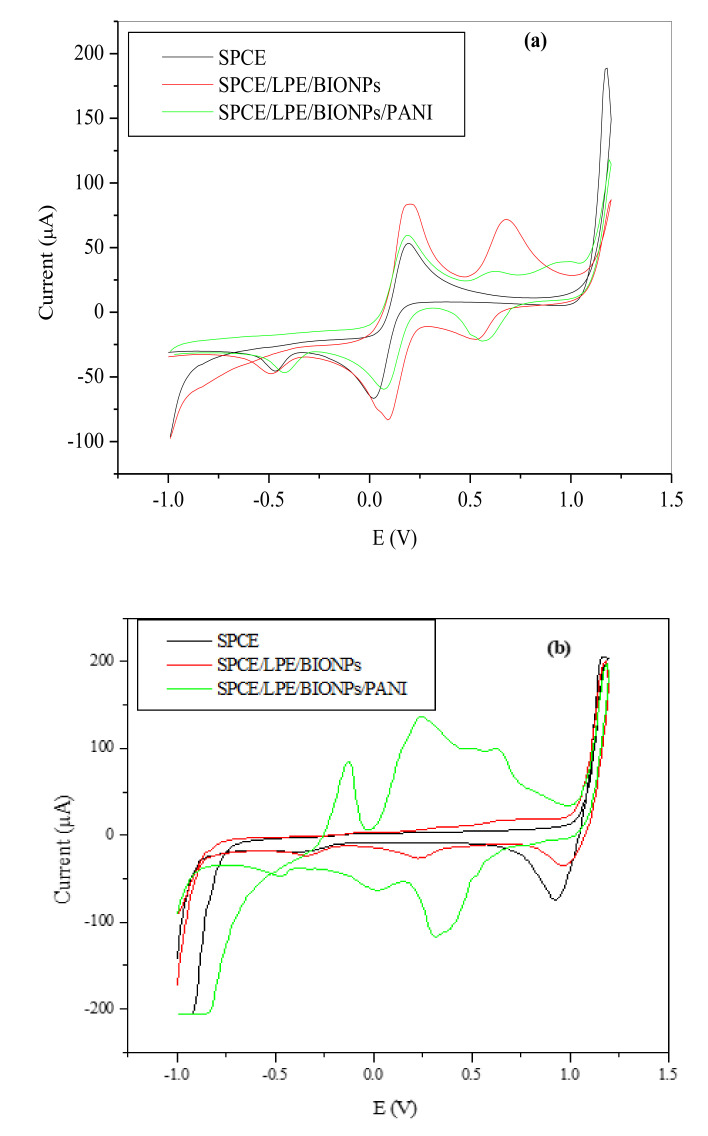
The comparative voltammogram of the SPCE, SPCE/LPE/BiONPs and SPCE/LPE/BiONPs/PANI in (**a**) 0.1 M HCl (scan rate = 0.1 V/s) and (**b**) probe (scan rate = 0.01 V/s). Potentials vs. Ag/AgCl.

It is expected that the blend of NPs with high surface area and high sensitivity, and less porous conductive PANI polymer will bring some enhancement in the electro-activity of the bare SPCE. These BIO NPs were mixed with conductive PANI polymer in approximately 2:1 ratio (BIO NPs: PANI) and evaluated for possible superior performance, relative to just BIO NPs as illustrated in Figure 6. In Table 2, the redox peak current was higher in SPCE/LPE/BIONPs than that of the SPCE/LPE/BIONPs/PANI though. This is likely due to the electrostatic repulsion between the negatively charged nitrogen ions in both probe and PANI. IN the absence of such repulsive force in the 0.1 M HCl electrolyte, the SPCE/LPE/BIONPs/PANI, gave a much higher current response with defined redox peaks than both the bare and SPCE/LPE/BIONPs.

### 3.5. Electrochemical Detection of Pb^2+^ Using SWV Technique

#### 3.5.1. Optimization

To obtain the best SWV results of the target Pb^2+^ analyte it was necessary to optimize the following sensing parameters, namely the pH, deposition time and potential, frequency and pulse amplitude.

##### 3.5.1.1. Choice of Supporting Electrolyte

**Figure 7:**
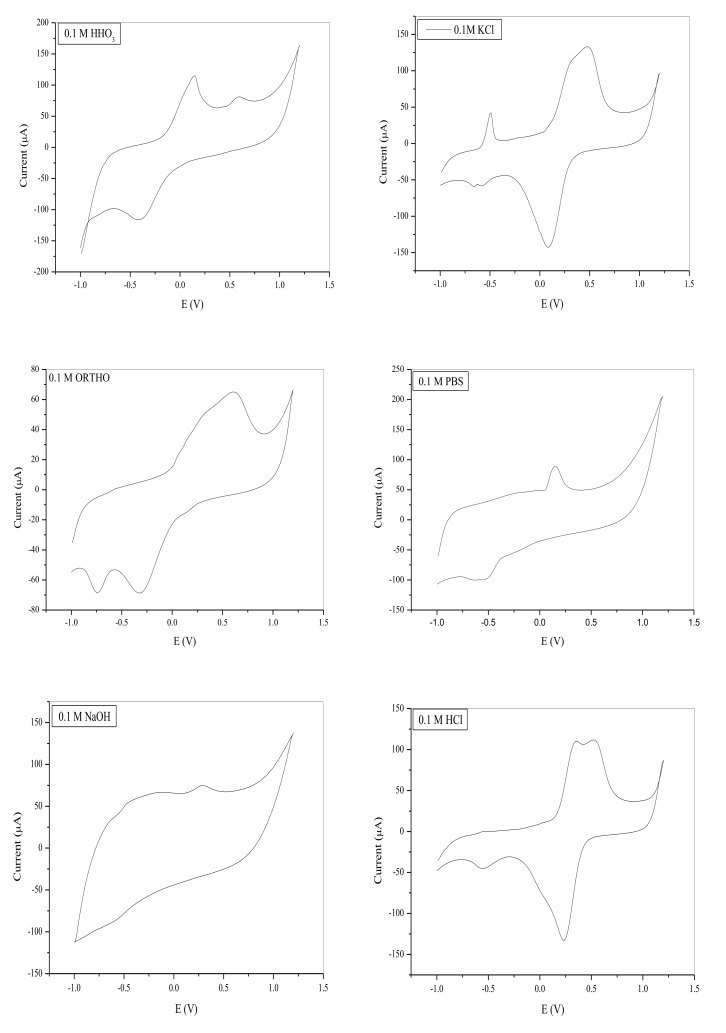
CV of the 0.1 M supporting electrolyte at the SPCE/OPE/LBIONPs, (scan rate = 0.1 V/s, Potentials vs. Ag/AgCl).

The importance of the best choice of supporting electrolyte has been commonly reported in the literature [70,71,72,73]. Hence, seven potential electrolytes were considered. These include (0.1 M) acetic acid (CH_3_COOH), nitric acid (HNO_3_), potassium chloride (KCl), ortho-phosphoric acid (H_3_PO_4_), sodium chloride (NaCl), phosphate buffer (PBS), and hydrogen chloride (HCl). H_2_SO_4_ was omitted for concerns of reacting with lead nitrate to form insoluble lead salt. The cyclic voltammetry was run using a common potential window of −1000 mV to 1200 mV, potential step fixed at 0.01 V at a scan rate of 0.1 V/s, as shown in Figure 7. Generally, the PANI based electrode exhibits dissimilar cyclic voltammetric response in different acid electrolytes which may be as a result of the diverse size and charge of acid [69].

Table 3 shows that all the electrolytes demonstrated redox ability with defined peaks except in acetic acid. The oxidation peak around 0.12–0.15 V observed in HNO_3_ and PBS typifies the conversion of leucoemeraldine base to emeraldine salt [40]. HNO_3_, ortho-phosphoric acid and NaOH demonstrated redox peaks that indicate the oxidation or reduction of either of the PANI peaks, but not both, which may undermine selectivity of the electrode, and more so at a lower peak current. HCl and KCl demonstrated similar current response, but at a higher scan rate, one of the characteristic peaks of PANI disappeared in KCl but remained in HCl. The implication is that, in HCl electrolyte, the electrode may be more selective towards target ions than in KCl electrolyte, even though the later appeared to have higher peak current response. Hence, HCl was chosen as a better electrolyte for this study.

###### Electrochemical Characterization of the SPCE Modified Electrodes in 0.1 M HCl Electrolyte

**Figure 8:**
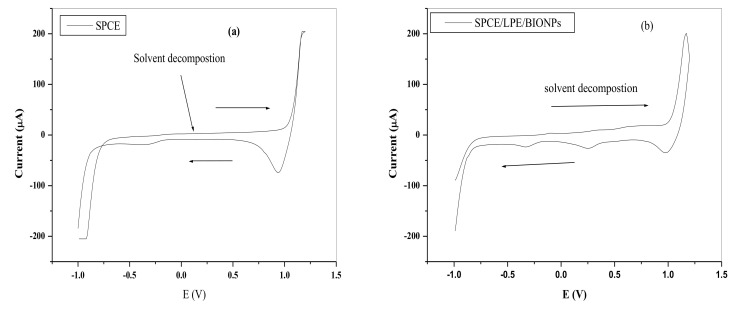

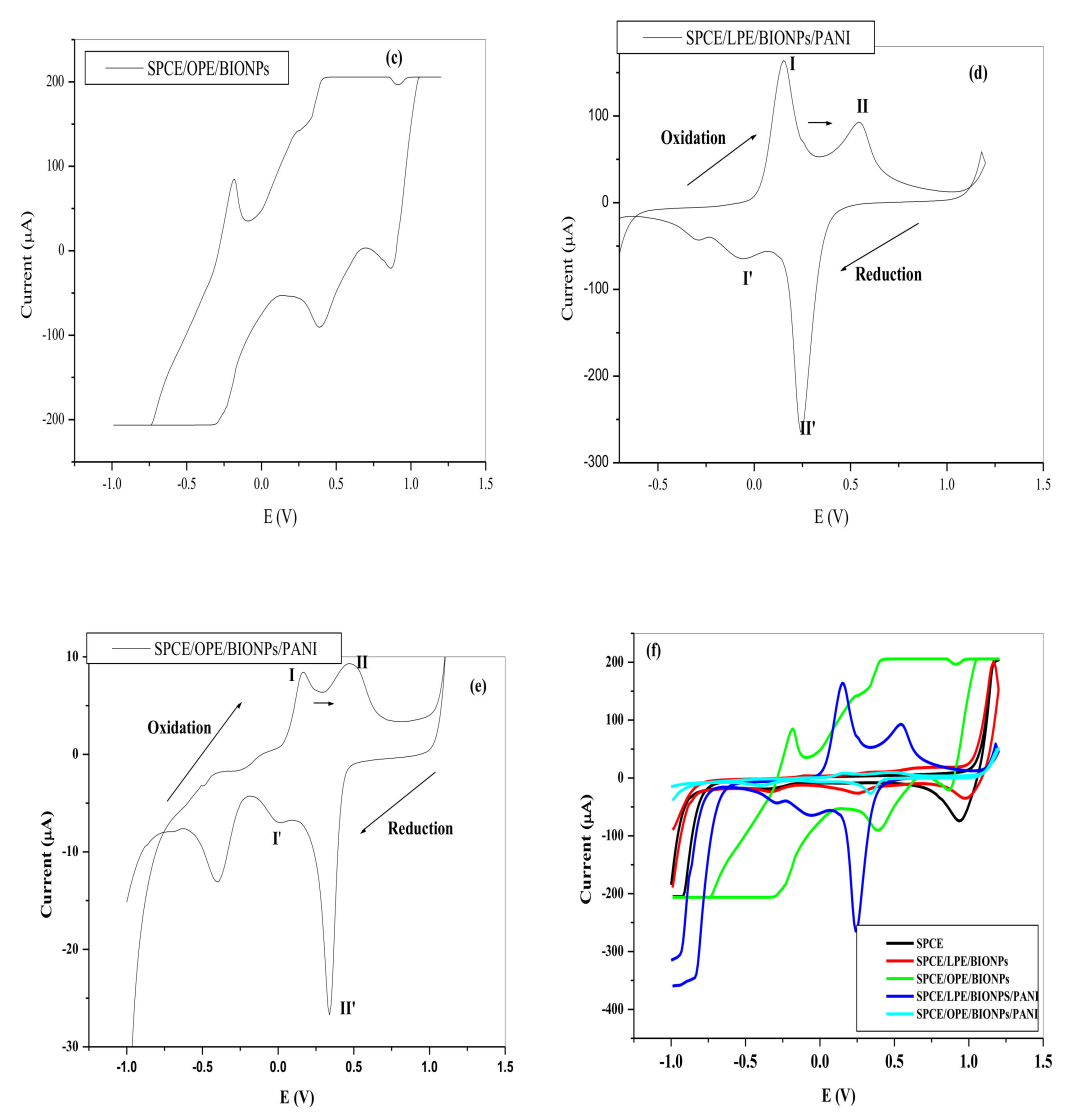
The CV of the (**a**) SPCE (**b**) SPCE/LPE/BiONPS (**c**) SPCE/OPE/BiONPs (**d**) SPCE/LPE/BiONPs/PANI (**e**) SPCE/OPE/BiONPs/PANI and (**f**) Comparative voltamogram of the electrodes in 0.1 M HCl, (scan rate = 0.1 V/s, pH = 1.0, Potentials vs. Ag/AgCl).

Figure 8 shows the cyclic voltammetry of the unmodified and modified BIONPs-PANI composites of the SPCE in 0.1 M HCl electrolyte solution. The electrochemical redox behaviour of the modified electrodes was investigated over the potential window of −1000 mV to 1200 mV. The forward and backward scan on the bare SPCE electrodes in the electrolyte showed only solvent decomposition current without any pronounced redox peaks. The SPCE/LPE/BIONPs, followed a similar trend but with a less pronounced reduction potential peak at around 0.23 V, which could be attributed to reduction of Cu^2+^ to Cu^0^. In SPCE/OPE/BIONPs, solvent decomposition currents were observed at more negative potential values than positive potential values.

The current response far outstripped the upper limit of the drop-sense current window resulting in voltammetry that looks similar to solvent decomposition at the central potential range. However, an oxidation peak was noticeable at around −0.2 V, which could be attributed to oxidation of Cu^0^ to Cu^2+^ salt [74]. At SPCE/LPE/BIONPs/PANI and SPCE/OPE/BIONPs/PANI also, solvent decomposition was observed at more positive or negative potentials. In the forward scan, two anodic peak currents designated as **I** and **II** were seen at about 0.14 V and 0.53 V for SPCE/LPE/BIONPs/PANI and 0.16 V and 0.48 V for SPCE/OPE/BIONPs/PANI, respectively (Figure 8d–e). In the backward scan, two pronounced reduction peaks designated as I’ and II’ were around −0.08 V and 0.24 V for SPCE/LPE/BIONPs/PANI and 0.00 V and 0.33 for SPCE/OPE/BIONPs/PANI, respectively. The redox peaks at I/I’ and II/II’ are similar to that of PANI reported in the previous works for SPCE and other bulk electrodes. The oxidation peaks at I and II in the forward scan are typical of to the conversion of leucoemeraldine base to emeraldine salt and the emeraldine salt to pernigraniline salt forms. While the backward scan peaks represented by I’ and II’’ are characteristic of the transformation of pernigraniline salt to emeraldine salt and emeraldine salt to leucoemeraldine base, respectively [40,75].

Typically, electrodes modified by conducting polymers, follow three processes which are: (1) a heterogeneous transfer of electrons between the electrode surface and the layer of the polymer; (2) electrons diffusions along the chain of the polymer, and (3) the solution species diffusions to the electrode [40,76,77,78,79]. In addition, changes in the kinetic properties for electrodes of various polyanilines (PANIs) is probable and it is basically dependent on synthesis conditions such as the nature of supporting electrolyte employed, electrosynthesis solution pH, the concentration of the monomer, and potential applied [40,77], which were all justified in the optimization.

**Figure 9:**
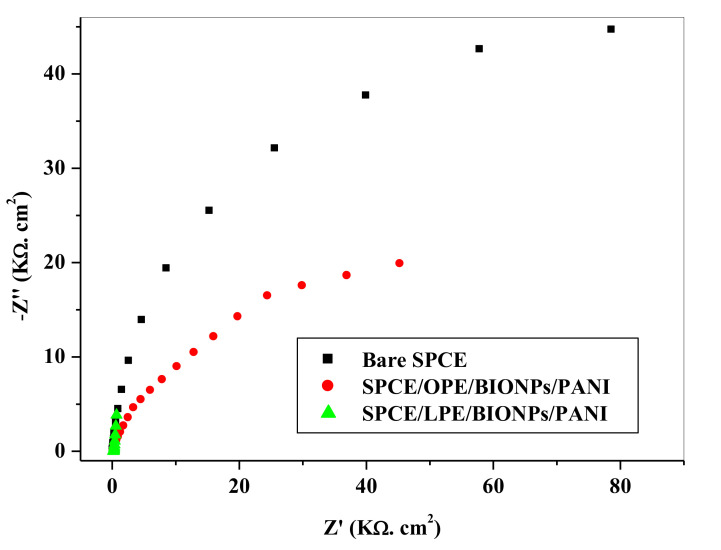
Electrochemical Impedance Spectroscopy of the Bare and the Modified Electrodes.

Electrochemical impedance is a successful technique for the study of the interfacial properties of electrode modified surface. The characteristic impedance of any electrode system is essentially dependent on the accumulative contribution of numerous parameters, namely (1) electrolyte resistance (R_s_), (2) charge transfer resistance (*R_ct_*) between the solution and the electrode surface, (3) Warburg element (Z*_w_*), and (4) double layer capacitance (*C_dl_*) (caused by the interface between the surface of the electrode and the solution). The engagement of a constant phase element in place of the capacitance is necessary in the optimization of the fit in the experiment, which is attributable to the deviation from idealist nature of the electrode [80,81]. The impedance complexity can be explained as the total of the real (Z*_rel_*) and the imaginary (Z*_w_*) element, originating from the cell resistance and capacitance. In addition, for the interest of providing further comprehensive report on the impedance of the SPCE modified electrode, an adapted Randles equivalent circuit fitting was preferred in fitting the measured results. Hence, the Randle equivalent circuit was employed in fitting the impedance data in Figure 9 with the given circuit parameters: solution resistance (R_s_), charge transfer resistance (R*_ct_*), the double layer capacitance (C*_dl_*), and Warburg impedance (Z*_w_*). The interfacial properties of the electrode were represented by the double layer capacitance and the charge transfer resistance, which are essentially regulated by the modification of the surface of the electrodes.

As noted in the CV in the 0.1 M HCl electrolyte solution, in the previous section, the presence of the nanocomposite on the SPCE surface drastically reduced the Rct values: 175 kΩ (SPCE) to 100 Ω (SPCE/LPE/BiONPs/PANI), equivalent to 99.94% reduction and 65 kΩ (SPCE/OPE/BiONPs/PANI) corresponding to 62.86% reduction (Figure 9). This development springs from the enhanced electroactive surface area and interfacial kinetics of the nanocomposites on the surface of the SPCE. An R*_ct_* of 100 Ω and 65 kΩ indicates a synergic blending of the CPE/BiONPs and PANI. The Nyquist plots data demonstrated that the modifying layers were effectively deployed onto the electrode surface.

##### 3.5.1.2. Effect of pH

**Figure 10:**
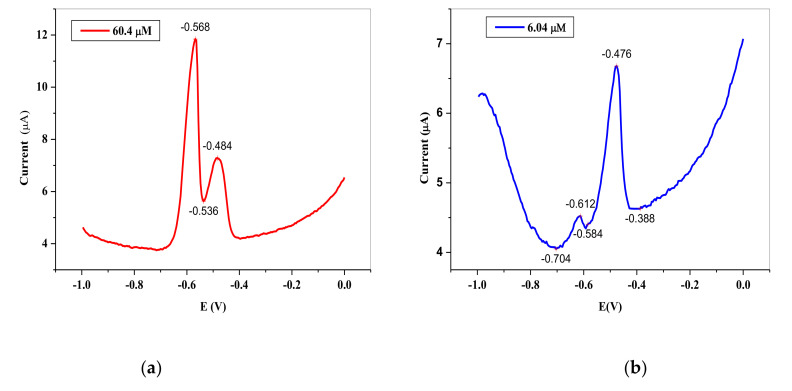

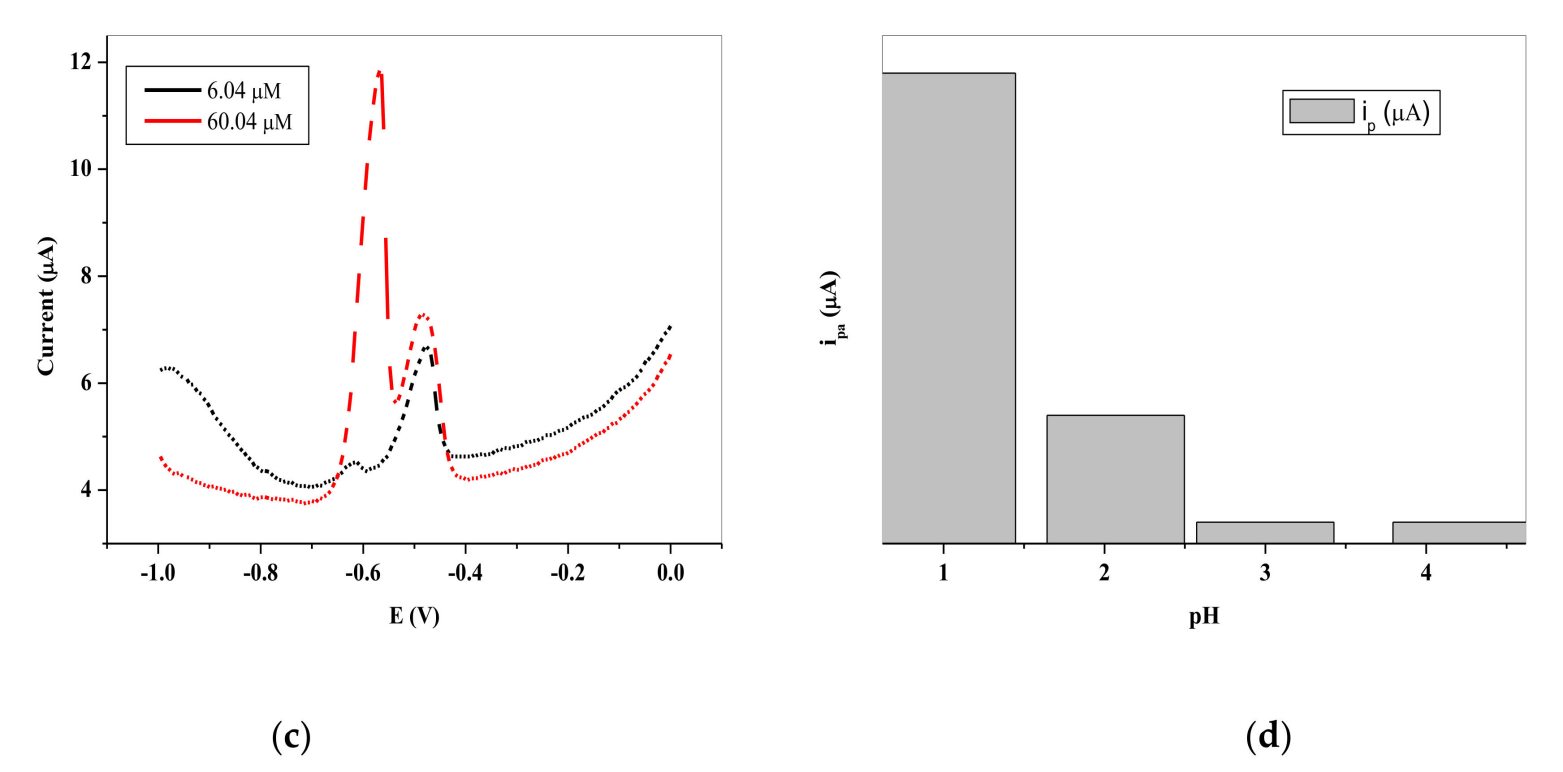
Typical SWV of different Pb^2+^ concentrations in 0.1 M HCl electrolyte on SPCE/OPE/BIONPs/PANI, (**a**) 6.04 µM, (**b**) 60.4 µM, (**c**) 6.04 µM and 60.4 µM (**d**). The influence of pH on the stripping peak current of 6.04 µM Pb2^+^, 1100 mV deposition potential, 30 s deposition time, (Potential vs. Ag/AgCl).

Figure 10a–c shows the current response in SWV mode of the different concentrations of Pb^2+^ solution in the 0.1 M HCl analyte at SPCE/OPE/BIONPs/PANI, deposition time of 30 s, and deposition potential of −1100 mV, and prior to using optimized conditions. The electrodes showed two stripping peaks at about −0.568 V and −0.476 V, indicative of two binding sites with different energy levels, as a result of the heterogeneity of SPCEs [82]. The site at −0.568 V, showed higher current response with increasing concentration of Pb^2+^ throughout the study, suggesting that it is the site with the higher energy site with strongest adsorption [83]. As seen and expected in the current response, the anodic stripping peak current increased with increasing Pb^2+^ concentration. However, there are other measuring parameters that could affect the current response apart from the concentration of the target analyte and one of them is the pH of the solution. The anodic current is favourably dependent on the target analyte solution. Hence, the effect of the pH of on the stripping current of the Pb ^2+^ was investigated over the acidic region as most literature reports indicate that the acid medium favours the electrochemical stripping of Pb^2+^, Cd^2+^, and Hg^2+^ [82]. Figure 10d shows the magnitude of the current response on SWV mode at various pH of the analyte solution. At pH of 1, the change in magnitude of the peak current (∆i_p_) was highest and hence was selected as the optimal pH for the electrochemical quantification of the Pb^2+^ analyte, which could be attributed to reduction in the H^+^ ions in solution that grow weaker the complexes established between the Pb^2+^ ions and amino groups [83]. This also agrees with other literature report [40]. Besides the steadily decreasing ∆i_p_ is also the concern of rise in background current with increasing pH.

##### 3.5.1.3. Effect of Deposition Time

**Figure 11:**
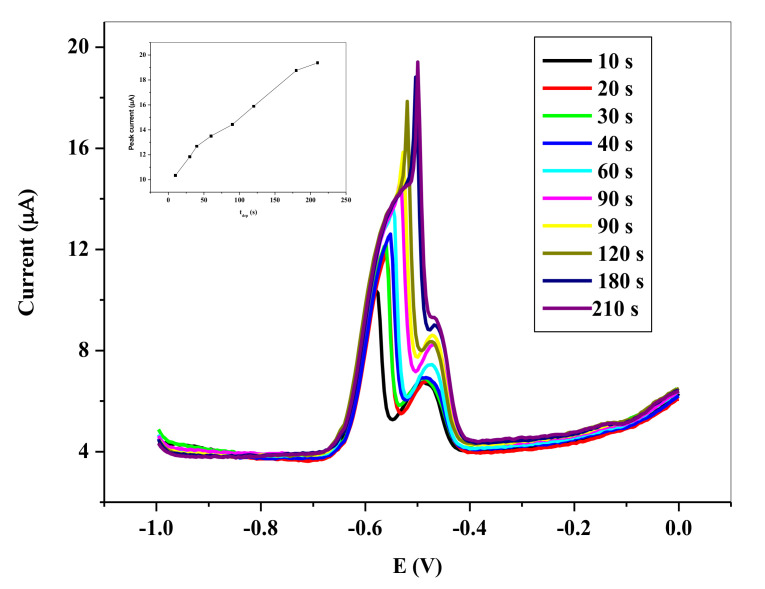
SWVs showing impact of deposition times on the stripping peak current of 6.0 × 10^−5^ M Pb^2+^ in 0.1 M HCl electrolyte on SPCE/OPE/BIONPs/PANI, 1100 mV deposition potential, 30 s deposition time, (Potential vs. Ag/AgCl).

Another important parameter optimized is the deposition time. To obtain the optimum deposition time, the SWVs were taken at different times, 10, 20, 30, 40, 60, 90, 120, 180, and 210 s, as shown in Figure 11**.** It is apparent that the anodic peak current continued to increase with increasing deposition time somewhat sharply until 180 s. Hence, 180 s was chosen as the optimum deposition time. This however is not to say that the highest peak current was obtained at that time, but indicates a saturating of the electrode surface, due to the electro-deposition of the Pb^0^.

##### 3.5.1.4. Effect of Deposition Potential

**Figure 12:**
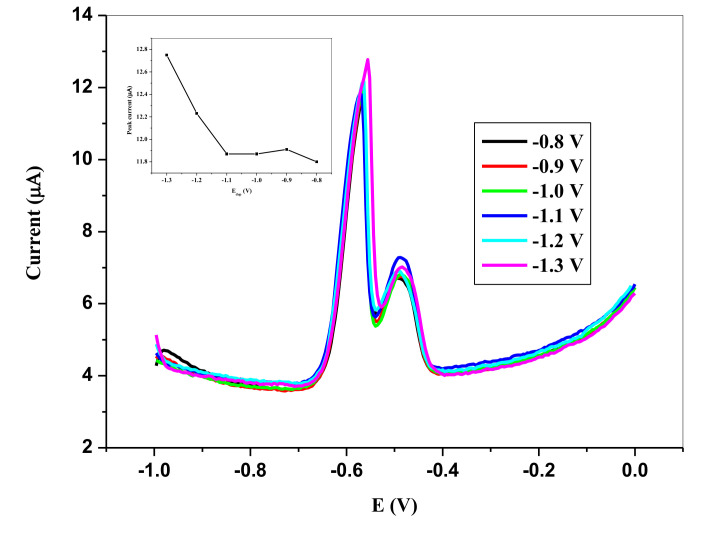
SWVs showing impact of deposition potential on the stripping peak current of 6.0 × 10^−5^ M Pb^2+^ in 0.1 M HCl electrolyte on SPCE/OPE/BIONPs/PANI, −800 mV to −1300 mV deposition potential, 180 s deposition time, (Potential vs. Ag/AgCl).

A potential range guided by other literature reports were chosen from −0.8 to −1.2 V and examined (Figure 12). Peak currents were observed at two stripping potentials, higher one at about −0.57 V and a second lower one at −0.49 V, due to the heterogeneity of the SPCE. The peak current increased with increasing negative potential till −1.3 V at both stripping potentials. It was necessary to keep the deposition potential at a moderate potential, to reduce the possible interference of the second lower peak and hence, 1.2 V was selected as the optimal deposition potential.

##### 3.5.1.5. Effect of Frequency and Square Wave Amplitude

**Figure 13:**
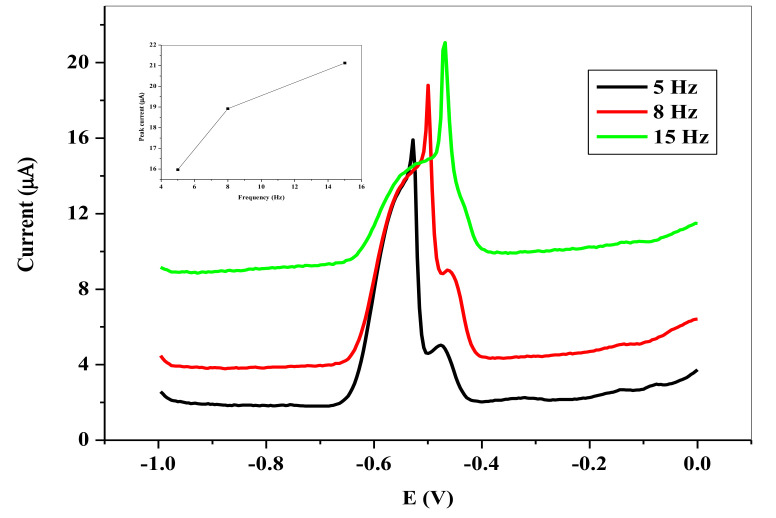
SWVs showing influence of frequency on the stripping peak current of 6.0 × 10^−5^ M Pb^2+^ in 0.1 M HCl electrolyte on SPCE/OPE/BIONPs/PANI, 1200 mV deposition potential, 180 s deposition time, (Potential vs. Ag/AgCl).

**Figure 14:**
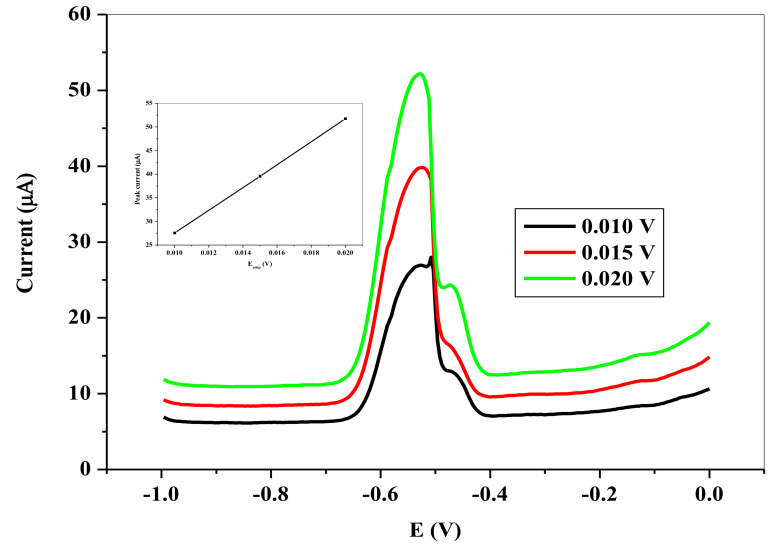
SWVs showing influence of pulse amplitude on the stripping peak current of 6.0 × 10^−5^ M Pb^2+^ in 0.1 M HCl electrolyte on SPCE/OPE/BIONPs/PANI, 1200 mV deposition potential, 180 s deposition time, (Potential vs. Ag/AgCl).

Figure 13 represents the dependence of the stripping peaks on the frequency of the voltammetry. At higher frequency, the anodic stripping peak currents increases with a skew to the potential. Unfortunately, the background also increases with this increase in the peak current. The difference between the SWV ***i_pa_*** for the frequency of 8 Hz and 15 Hz is small compared to the background current generated. In actual sense, the ∆*i_p_* was higher at the frequency of 8 Hz, hence it was used as the optimum frequency. Figure 14 shows the dependence of the ***i_pa_*** on the SW amplitude. Similar to the case of the frequency, the SWV ***i_pa_*** continued to rise with attendant increase in the background, as the amplitude increased. However, an appropriate SW amplitude of 25 mV was used as the optimal amplitude in the sequence of the experiment.

### 3.6. Calibration of Sensors and Detection of Limit Determination

Calibration plots were generated from the square wave voltammograms of the SPCEs and examined under the conditions chosen and summarized in Table 4. With the aim of evaluating the ability of the SPCEs to detect the smallest introduction of the metal inorganic pollutant, the curves were generated by spiking a fixed volume (10 mL) of the 0.1 M HCl electrolyte solution with small amount of the standard laboratory prepared concentrations of the target metal ion solution.

**Figure 15:**
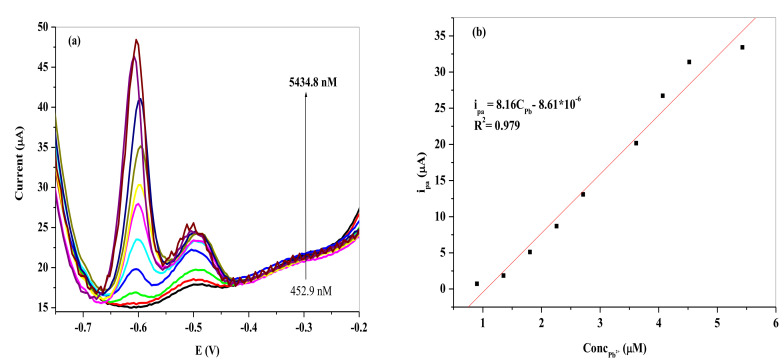
(**a**) SWVs of different concentrations of Pb^2+^ on SPCE/LPE/BIONPs/PANI. (**b**) Calibration curves of the peak current vs. different concentrations of Pb^2+^. (Potential vs. Ag/AgCl).

**Figure 16:**
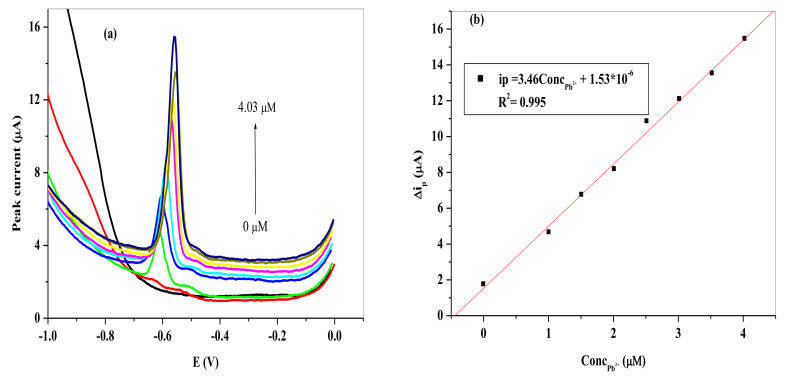
(**a**) SWVs of different concentrations of Pb^2+^ on SPCE/OPE/BIONPs/PANI. (**b**) Calibration curves of the peak current vs. different concentrations of Pb^2+^. Potential vs. Ag/AgCl).

Figure 15 and Figure 16 show the SWV of the different Pb^2+^ concentrations (a) and the resultant calibration curves (b). The i_pa_ increased linearly with increasing Pb^2+^ concentration on the two SPCEs represented by the following equation:

(1) $$i_{\mathrm{pa}}=8.16{{\mathrm{Conc}}_{\mathrm{Pb}}}^{2+}-8.61\times 10^{-6}\;(R^{2}\;=\;0.979)\;\mathrm{for}\;\mathrm{SPCE}/\mathrm{LPE}/\mathrm{BIONPs}/\mathrm{PANI}$$

(2) $$i_{\mathrm{pa}}=8.17{{\mathrm{Conc}}_{\mathrm{Pb}}}^{2+}-1.84\times 10^{-6}\;(R^{2}\;=\;0.997)\;\mathrm{for}\;\mathrm{SPCE}/\mathrm{OPE}/\mathrm{BIONPs}/\mathrm{PANI}$$

The limit of detection and quantification were evaluated based on the relation in Equations (3) and (4)

LOD = 3σ/S(3)

LOQ = 10σ/S(4)

where σ is the standard deviation of 6 consecutive SWV blank and S is the calibration curve slope.

The LOD and LOQ were computed using Equations (1) and (2) for Pb^2+^ to be 1.49 nM (0.494 ppb) and 4.90 nM at SPCE/LPE/BIONPs/PANI (*n* = 9), respectively. While the LOD and LOQ computed for Pb^2+^ at SPCE/OPE/BIONPs/PANI (*n* = 8) were 8.40 nM (2.79 ppb) and 28.00 nM (8.91 ppb), respectively. Apparently the SPCE/LPE/BIONPs/PANI demonstrated superior electrochemical sensitivity with a broader linear range than the SPCE/OPE/BIONPs/PANI towards Pb^2+^, however with lower R^2^ value. The LODs for the SPCEs are well above the limit for Pb^2+^ concentration in drinking water by WHO (10 ppb) and US-EPA (15 ppb).

Table 5 shows the comparison of the electrochemical performance of carbon-based electrodes in the detection of Pb^2+^, and demonstrates the applicability of the SPCE/LPE/BIONPs/PANI and SPCE/OPE/BIONPs/PANI in environmental monitoring of Pb^2+^ in water. 

### 3.7. Evaluation of the Precision of the SPCEs

**Figure 17:**
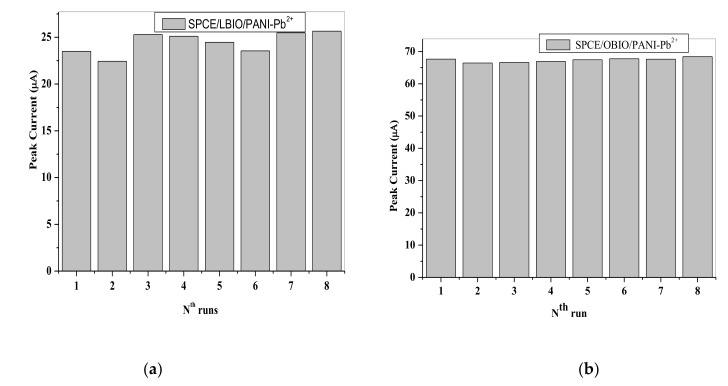
SWV anodic peak current magnitude for eight consecutive runs of 6.04 µM of Pb^2+^ at (**a**) SPCE/LPE/BIONPs/PANI. (**b**) SPCE/OPE/BIONPs/PANI.

Precision of the SPCEs were evaluated upon measuring the stripping responses of the samples (*n* = 8) having Pb^2+^ concentration as shown in Figure 17. The relative standard deviations (RSD) are presented computed at SPCE/LPE/BIONPs/PANI and SPCE/OPE/BIONPs/PANI are 4.76% and 0.98%, respectively. These very low RSD show that electrodes were highly stable and could be used in environmental monitoring of these HMs. It is reported that the stability is a result of the presence of PANI, which covers the surface of the NPs, thereby blocking surface active compound present in the bulk solution.

### 3.8. Real Sample Analysis

**Figure 18:**
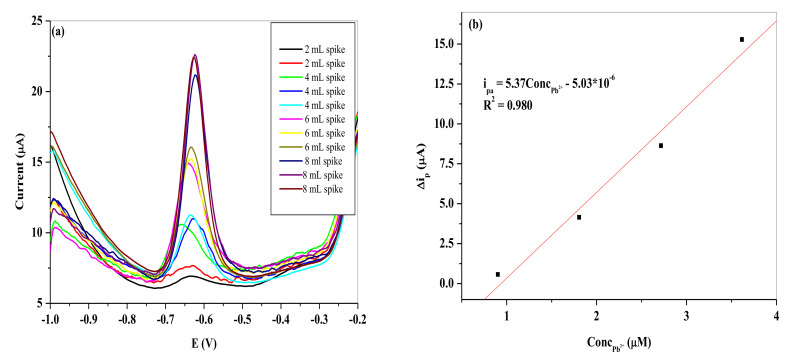
Represent **(a)** the SWV of the different spikes with Pb^2+^ concentration and (**b**) the resultant calibration curve at SPCE/LPE/BiONPs/PANI at optimum conditions. (Potential vs. Ag/AgCl).

**Figure 19:**
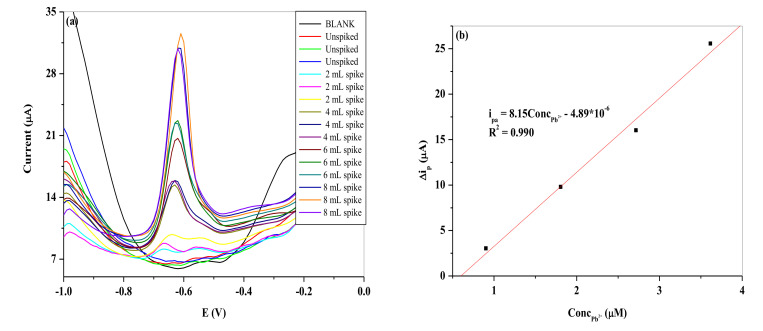
Represent (**a**) the SWV of the different spikes with Pb^2+^ concentration and (**b**) the resultant calibration curve at SPCE/OPE/BiONPs/PANI at optimum conditions. (Potential vs. Ag/AgCl).

**Figure 20:**
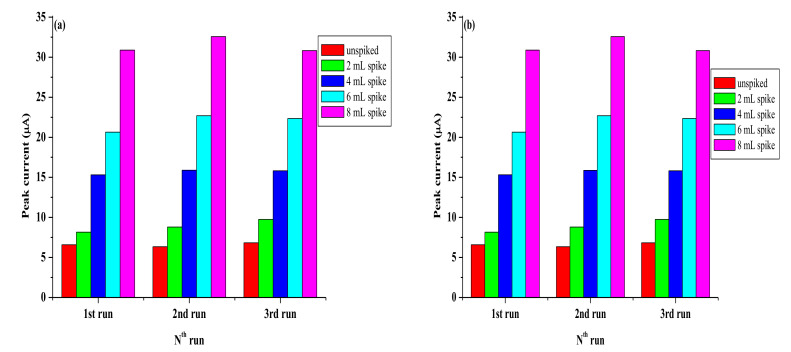
The precision of the electrodes towards the Pb^2+^ analyte concentration at (**a**) SPCE/LPE/BiONPs/PANI and (**b**) SPCE/OPE/BiONPs/PANI.

The electrochemical sensor fabricated was subjected to detection of Pb^2+^ in real water sample as shown in Figure 18 and Figure 19. The water was collected from a crocodile river in South Africa with the coordinate shown in Table 6. The experiment was done in triplicates (*n* = 3), with the precision typified in Figure 20 and the average of the peak current responses was used for the calibration plots. The slopes of the calibration plots are represented by the following equation on the two electrodes:

i_pa_ = 5.37 [Pb^2+^] − 5.03 × 10^−6^ SPCE/LPE/BIONPs/PANI (5)

i_pa_ = 8.15 [Pb^2+^] − 4.89 × 10^−6^ SPCE/OPE/BIONPs/PANI (6)

The results as shown in Table 7 show a good recovery rate of 104.32% and 103.32% for SPCE/LPE/BIONPs/PANI, and SPCE/OPE/BIONPs/PANI, respectively, when the river water sample was spiked with 3.6 µM of Pb^2+^ concentration. The average recovery rates for the two electrodes are, however, 94.25% and 96.70%, in that same order. The t-test also shows a *p*-value at 95% confidence level that is less than 0.05 for both calibrations from the two electrodes (Figure 18b and Figure 19b), showing high precision of the calibration coefficients despite the number of different concentrations (spikes) considered. Using SWV, the fabricated sensor detected Pb^2+^ concentration of 0.727 µM (240.78 ± 0.18 ppb) and 0.683 µM (226.21 ± 0.29 ppb) at SPCE/LPE/BIONPs/PANI, and SPCE/OPE/BIONPs/PANI, respectively. The results are relatively comparable. However, the ICP-MS analysis result showed the concentration of Pb^2+^ in the river was below the instrument’s limit of detection. The ability of the engineered electrodes to detect Pb^2+^ in this river water samples, hence underscores the superior sensitivity and selectivity of the electrochemical techniques employed in environmental monitoring strategy. Worthy to mention is that the Pb^2+^ concentration detected in the river samples is well above the recommended unit for safe drinking water by WHO but well below the standard set by other environmental agencies for Pb^2+^ in sea water. It could hence be argued that run off waters from the agricultural and mining activities close to the site was responsible for the high concentration of Pb^2+^ in the fresh river water sampled.

### 3.9. Interference Studies

The selectivity of the fabricated SPCE/OPE/BIONPs/PANI towards the target analyte was examined in the presence of six other metals commonly associated with, Pb^2+^, at equal molar concentrations and presented in Table 8 below. The results showed that the electrode was highly selective towards the target analyte in the presence of these metals. However, Co^2+^ reduced the stripping peak current of Pb^2+^ by 17.67%. The SPCE/LPE/BIONPs/PANI was so susceptible to interference of these metals that we consider further details into that a future work to be carried out.

## 4. Conclusions

A common environmental citrus waste peel of lemon and orange were successfully employed in the green synthesis of ZnO and CuO blends to form bioxide of BiONPs in an alkaline medium. The synthesized NPs were characterized using UV-Vis, SEM, and FTIR, which showed basic spectroscopic properties that were consistent with the monoxide components of the BiONPs. This posits that nanotechnology could be used to recycle organic environmental wastes, hitherto poorly managed. Citrus peels extracts mediated BiONPs/PANI polymer were exploited in the modification of the SPCE and an improved electrochemical response relative to the unmodified SPCE and SPCE/PANI electrodes using CV and EIS characterization was realized. The presence of the nanocomposite on the SPCE surface drastically reduced the R*_ct_* value of the bare SPCE from 175 kΩ to 100 Ω at SPCE/LPE/BiONPs/PANI, which is equivalent to 99.94% reduction, and 175 kΩ (SPCE) to 65 kΩ at SPCE/OPE/BiONPs/PANI, corresponding to 62.86% reduction. A detection limit of 0.494 ppb and 1.647 were obtained at SPCE/LPE/BiONPs/PANI, respectively, while a detection and quantification limit of 2.79 ppb and 8.91 ppb, respectively, were derived from SPCE/OPE/BiONPs/PANI. The RSD computed for both electrodes, were less than 5%. The SPCE/OPE/BiONPs/PANI showed mostly negligible reduction in peak current in the presence of interfering metals. These further underscore the sensitivity, stability, selectivity and the applicability of the tools and technique used. An optimal potential voltage of −1.2 V for 180 s in 0.1 M HCl supporting electrolyte was employed. The two working electrodes developed showed good applicability in real river water sample analysis. Both electrodes showed limits of detection for Pb^2+^ that are far below the standard recommended by WHO and US-EPA in drinking water and comparable to other reported works. Therefore, the employed technique and electrodes modified with eco-friendly, simple and low-cost synthesized nanocomposites, could be employed as a good on the site, low cost and easy to operate strategy in the environmental sensing application.

## Figures and Tables

**Table 1:** Ultra-pure water quality (type 1) used.

Water Resistivity MΩ cm @ 25 °C	Ionic Concentration (µg/L)
18.2	<1
17.0	<2
16.0	<3
15.0	<5
10.0	<10

**Table 2:** The peak summary for the comparative voltammogram of the SPCE, SPCE/LPE/BiONPs and SPCE/LPE/BiONPs/PANI in the probe.

ELECTRODES	E_pa_ (V)	I_pa_ (µA)	E_pc_ (V)	I_pc_ (µA)	∆E_p_ (V)	$\frac{i_{pa}}{i_{pc}}$
SPCE	0.190	53.379	0.020	−66.229	0.17	0.806
SPCE/PANI	0.220	95.620	0.020	−113.910	0.12	0.840
SPCE/LPE/BIONPs	0.192	83.587	0.0925	−83.324	0.10	1.003
SPCE/LPE/BIONPs/PANI	0.182	59.421	0.0765	−60.181	0.11	0.987

**Table 3:** Peak properties of the investigated electrolyte.

Electrolyte	I_p_ (µA)	Potential Window	Curve Coverage Area
Acetic acid	-	−1.0 to 1.2	74.444
Nitric acid	80.164	−1.0 to 1.2	173.414
Potassium chloride	134.245	−1.0 to 1.2	169.226
Hydrogen chloride	110.445	−1.0 to 1.2	140.949
Phosphate Buffer Solution	-	−1.0 to 1.2	192.919
Ortho-phosphoric acid	65.245	−1.0 to 1.2	111.044
Sodium hydroxide	73.164	−1.0 to 1.2	203.585

**Table 4:** Optimal conditions for the electrochemical deposition of Pb^2+^ analyte.

Optimal Conditions	
Deposition time	180 s
Electrolyte	0.1 M HCL
Deposition potential	−1.2 V
pH	1.01
Frequency	8 Hz
Potential Step	4 mV
Pulse Amplitude	25 mV

**Table 5:** The comparison of LODs and LOQs of the Pb^2+^ analytes at the SPCE/LPE/BiONPs/PANI and SPCE/OPE/BiONPs/PANI, with other reported modified electrodes, given optimum conditions. Potential vs. Ag/AgCl.

Electrodes	Sensitivity (µA/µM)	STD. DEV.	LOD (ppb)	LOQ (ppb)	Linear Range (µM)	Method Analysis	Deposition Time (s)	Electrolyte/ pH	Reference
BiF-SPCE	-	-	0.008	0.027	0–0.1	AdDPSV	180	0.01 M Ammonium Buffer pH = 9.2	[84]
Bare-SPCE	-	-	2.5	8.33	6.3- 24	DPASV	150	0.1 M HCl	[81]
CAL-SPCEs	-	-	5.0	16.67	0–0.01	DPASV	5	1.0 M ammonia buffer at pH 11.5	[85]
AuNP/ERGO-SPCE	0.157	-	0.11	0.36	0.002–0.014	DPASV	300	0.02 M HCl	[86]
Cr (III) oxide -SPCE	-	-	3	10	0.03–2.42	SWASV	100		[87]
Mercury film SPE *	0.33	-	1.8	6.00	0.029–0.30	SWASV	120	0.6 M NaCl pH 8	[37]
DPTGCE	-	-	0.695	2.32	11–45	SWASV	120	0.1 M HCl pH 3	[88]
GCE/SWCNTs/BE	23.983	-	33.1	110.33	-	DPASV	120	0.1 M acetate buffer pH 4.5	[89]
AuNPs-SPCE	0.376	-	4.62	15.54	5–25	SWASV	-	0.2 M acetate buffer	[90]
SPCNFEs	0.1	-	2.8	9.33	2–100	DPASV	180	acetic acid/acetate buffer pH 4.5	[91]
MoS_2_/rGO-GCE	50.80	-	1.59	5.30	16.55–264.8	SWASV	120	0.1 M NH_4_Cl-HCl pH 4.0	[92]
Cr-CPE	18.75	-	3	10	0.03–2.42	SWASV	100	2 M acetate buffer, pH 5	[87]
SPCE/LPE/BIONPs/PANI	8.16	0.004	0.49	1.47	0.45–5.43	SWV	180	0.1 M HCl pH = 1	This work
SPCE/OPE/BIONPs/PANI	3.46	0.029	2.79	8.91	0–4.03	SWV	180		This work

DPASV: differential pulse anodic stripping voltammetry. SWASV: square wave anodic stripping voltammetry; AdDPSV: Adsorptive differential pulse anodic stripping voltammetry, *AuNP* gold nanoparticles, *ERGO* electroreduced graphene oxide. CAL: calixarenes BiF: Bismuth Film, MB: methylene blue, PANI-PDTDA: Polyaniline-poly (2,2′-dithiodianiline); *: Simultaneous detection on modified SPE electrodes, DPTGCE: 1-dodecanoyl-3-phenylthiourea glassy carbon electrode; GCE/SWCNTs/BE: Glassy carbon electrode –Single walled carbon nanotubes biomass electrode; SPCNFEs: Screen-printed carbon nanofiber electrodes; MoS_2_/rGO-GCE: Molybdenum dichalcogenides reduced graphene oxide glassy carbon electrode; Cr-CPE: Chromium (III) oxide modified carbon paste electrode.

**Table 6:** The coordinates of the Crocodile River Water Sampling Point.

Sampling Point	Coordinates	Site ID
1	25° 40′ 52.6 S 27° 48′ 12.6 E	Agriculture/Mining

**Table 7:** The calibration coordinates, working range, LOD, recovery rate and RSD data for the electrodes.

SPCEs	R^2^	a ± S_a_	b ± S_b_ (10^−6^)	Working Range (µM)	LOD (µM)	Amount Added (µM)	Amount Found (µM)	RSD (%)	*t_a_-Stat*	P_a_-Value
LPE/BIONPs/PANI	0.98	5.37 ± 0.18	5.03 ± 0.68	0.9–3.62	0.73	3.62	3.74 (104.32%)	3.6	9.782	0.01
LPE/BIONPs/PANI	0.99	8.15 ± 0.29	−4.9 ± 0.72	0.9–3.62	0.68	3.62	3.71 (103.32%)	3.1	14.051	0.005

**Table 8:** The interference of other metals to the simultaneous detection of the three-target analyte at SPCE/OPE/BiONPs/PANI, given optimum conditions. (Potential vs. Ag/AgCl).

Interference	Pb^2+^_RECOVERED_
Mg^2+^	99.9999
Cu^2+^	100
Co^2+^	82.33
Fe^2+^	100
Zn^2+^	98.07
Ni^2+^	100

## Data Availability

Data for the study is available upon request from the authors.
